# New Forms of Quantum Value Indefiniteness Suggest That Incompatible Views on Contexts Are Epistemic

**DOI:** 10.3390/e20060406

**Published:** 2018-05-24

**Authors:** Karl Svozil

**Affiliations:** Institute for Theoretical Physics, Vienna University of Technology, Wiedner Hauptstrasse 8-10/136, 1040 Vienna, Austria; svozil@tuwien.ac.at

**Keywords:** quantum mechanics, Gleason theorem, Kochen–Specker theorem, Born rule

## Abstract

Extensions of the Kochen–Specker theorem use quantum logics whose classical interpretation suggests a true-implies-value indefiniteness property. This can be interpreted as an indication that any view of a quantum state beyond a single context is epistemic. A remark by Gleason about the ad hoc construction of probability measures in Hilbert spaces as a result of the Pythagorean property of vector components is interpreted platonically. Unless there is a total match between preparation and measurement contexts, information about the former from the latter is not ontic, but epistemic. This is corroborated by configurations of observables and contexts with a truth-implies-value indefiniteness property.

## 1. Quantum Contexts as Views on States

Contexts arise naturally in quantum mechanics: they correspond to the “greatest classical subdomains within the expanse of conceivable quantum propositions:” for all empirical matters, every observable within a particular fixed context can be assumed classical with respect to and relative to that context. Therefore, according to Gleason [1], it appears prudent to assume that classical probabilities should be applicable to such classical mini-universes; and in particular, when considering observables within a given context. Gleason formalized this in terms of frame functions and proceeded to show how the quantum probabilities, in particular, the Born rule, can be “stitched together” from these classical bits and pieces. This paper can be seen as a prolegomenon to this approach; and as a contribution to the ongoing search for its semantics.

Formally, the concept of context can be exposed in two ways: one is in terms of “largest possible” sets of orthogonal pure states; that is, in terms of (unit) vectors and their linear spans. Another one is by maximal operators and the perpendicular projection operators in their non-degenerate spectral decomposition.

Let us start by supposing that contexts can be represented by orthonormal bases of Hilbert space. Due to the spectral theorem, this immediately gives rise to an equivalent conception of context: that as a maximal observable, which is formed by some (non-degenerate) spectral sum of the mutually orthogonal perpendicular projection operators corresponding to the basis states. This is just the expression of the dual role of perpendicular projection operators in quantum mechanics: they represent both pure states, as well as observable bits; that is, elementary yes-no propositions.

For the sake of an elementary example, suppose one is dealing with (lossless) electron spin state (or photon polarization) measurements. As there are two outcomes, the associated Hilbert space is two-dimensional. The two outcomes can be identified with two arbitrary orthogonal normalized vectors therein, forming an orthonormal basis. Suppose, for the sake of further simplicity, that we parametrize this basis to be the standard Cartesian basis in two-dimensional Hilbert space, its two vectors being (Equation (1.8), [2]) $|0\rangle ={\left(\begin{array}{c} 1,0 \end{array}\right)}^{⊺}$ and $|1\rangle ={\left(\begin{array}{c} 0,1 \end{array}\right)}^{⊺}$, where the superscript symbol “⊺” indicates transposition. Their dyadic products $E_{0}=\left|0\rangle \langle 0\right|={\left(\begin{array}{c} 1,0 \end{array}\right)}^{⊺}⊗\left(\begin{array}{c} 1,0 \end{array}\right)=\left(\begin{array}{cc} 1 & 0\\ 0 & 0 \end{array}\right)$, $E_{1}=\left(\begin{array}{cc} 0 & 0\\ 0 & 1 \end{array}\right)$ form the corresponding (mutually) orthogonal perpendicular projection operators. These contexts can be either represented in terms of vectors, like $C=\left\{|0\rangle ,|1\rangle \right\}$, or in terms of perpendicular projection operators, like $C=\left\{E_{0},E_{1}\right\}$.

Any two distinct numbers $\lambda_{0}\neq \lambda_{1}$ define a maximal operator through the “weighted” spectral sum:

(1) $$A=\lambda_{0}E_{0}+\lambda_{1}E_{1}=\lambda_{0}\left|0\rangle \langle 0\right|+\lambda_{1}\left|1\rangle \langle 1\right|=\left(\begin{array}{cc} \lambda_{0} & 0\\ 0 & \lambda_{1} \end{array}\right).$$

The term “maximal” refers to the fact that A “spans” a “classical sub-universe” of mutually commuting operators through variations of $f\left(A\right)=f\left(\lambda_{0}\right)E_{0}+f\left(\lambda_{1}\right)E_{1}$, where f:R↦R represents some real valued polynomial or function of a single real argument (§84, Theorems 1 and 2, p. 171, [3]). In particular, this includes the context $C=\left\{E_{0},E_{1}\right\}$ through the two binary functions $f_{i}\left(\lambda_{j}\right)=\delta_{ij}$, with i,j∈{0,1}.

## 2. Probabilities on Contexts in Quantum Mechanics

Let us concentrate on probabilities next. As already mentioned, Gleason [1] observed that classical observables should obey classical probabilities (this should be the same for Bayesian and frequentist approaches). Can we, therefore, hope for the existence of some “Realding”, that is some global ontology, some enlarged panorama of “real physical properties”, behind these stitched probabilities? As it turns out, relative to reasonable assumptions and the absence of exotic options, this is futile.

Formally, this issue can be rephrased by recalling that the main formal entities of quantum mechanics are all based on Hilbert space; that is, on vectors, as well as their relative position and permutations. A pure state represented as a vector |ψ〉 can be conveniently parameterized or encoded by coordinates referring to the respective bases. Because of their convenience, one chooses orthonormal bases, that is contexts, for such a parametrization. Why is convenience important? Because, as has been noted earlier, in finite dimensions *D*, any such context $C\equiv \{|e_{1}\rangle ,|e_{2}\rangle ,\ldots ,|e_{D}\rangle \}$ can also be interpreted as a maximal set of co-measurable propositions $C\equiv \{E_{1},E_{2},\ldots ,E_{D}\}$ with $E_{i}=|e_{i}\rangle \langle e_{i}|$, 1≤i≤D, as the latter refers to a complete system of orthogonal perpendicular projections, which are a resolution of the identity operator $I_{D}=\sum_{i=1}^{D}E_{i}$. For any such context, classical Kolmogorov probability theory requires the probabilities *P* to satisfy the following axioms:

**A1**  probabilities are real-valued and non-negative: $P(E_{i})\in R$, and $P(E_{i})\geq 0$ for all $E_{i}\in C$, or, equivalently, 1≤i≤D;
**A2**  probabilities of mutually-exclusive observables within contexts are additive: $P\left(\sum_{i=1}^{k\leq D}E_{i}\right)=\sum_{i=1}^{k\leq D}P\left(E_{i}\right);$
**A3**  probabilities within one context add up to one: $P\left(I_{D}\right)=P\left(\sum_{i=1}^{D}E_{i}\right)=1$.

How can probabilities $P_{\psi }\left(E\right)$ of propositions formalized by perpendicular projection operators (or, more generally, observables whose spectral sums contain such propositions) on given states |ψ〉 be formed that adhere to these axioms? As already Gleason pointed out in the second paragraph of (Section 1, p. 885, [1]), there is an ad hoc way to obtain a probability measure on Hilbert spaces: a vector |ψ〉 can be “viewed” through a “probing context” C as follows:

(i) For each closed subspace spanned by the vectors $|e_{i}\rangle$ in the context C, take the projection $E_{i}|\psi \rangle$ of |ψ〉 onto $|e_{i}\rangle$.
(ii) Take the absolute square of the length (norm) of this projection and identify it with the probability $P_{\psi }\left(E_{i}\right)$ of finding the quantum system that is in state |ψ〉 to be in state $|e_{i}\rangle$; that is (the symbol “†” stands for the Hermitian adjoint):
  (2) $$\begin{array}{c} P_{\psi }\left(E_{i}\right)={\left(E_{i}|\psi \rangle \right)}^{†}E_{i}|\psi \rangle =\langle \psi |E_{i}^{†}E_{i}|\psi \rangle \\ =\langle \psi |e_{i}\rangle \underset{=1}{\underset{︸}{\langle e_{i}|e_{i}\rangle }}\langle e_{i}|\psi \rangle =\langle \psi |e_{i}\rangle \langle e_{i}|\psi \rangle ={∥\langle e_{i}|\psi \rangle ∥}^{2}. \end{array}$$

**Figure 1:**
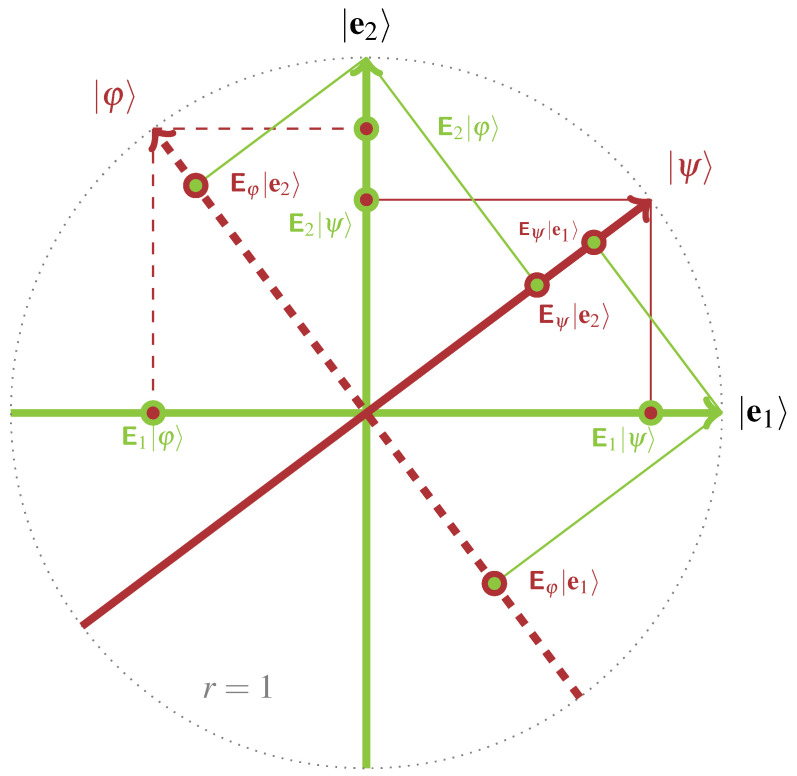
An orthonormal basis forming a context $C=\{|e_{1}\rangle ,|e_{2}\rangle \}$ represents a frame of reference from which a “view” on a state |ψ〉 can be obtained. Formally, if the vectors |ψ〉 and |φ〉 are normalized, such that 〈ψ|ψ〉=〈φ|φ〉=1, then the absolute square of the length (norm) of the projections $E_{1}\left|\psi \rangle =\right|e_{1}\rangle \langle e_{1}|\psi \rangle$ and $E_{2}\left|\psi \rangle =\right|e_{2}\rangle \langle e_{2}|\psi \rangle$, as well as $E_{1}\left|\varphi \rangle =\right|e_{1}\rangle \langle e_{1}|\varphi \rangle$ and $E_{2}\left|\varphi \rangle =\right|e_{2}\rangle \langle e_{2}|\varphi \rangle$ adds up to one. Conversely, a second context $C^{\prime }=\{|\psi \rangle ,|\varphi \rangle \}$ grants a frame of reference from which a “view” on the first context C can be obtained.

Because of the mutual orthogonality of the elements in the context C, the Pythagorean theorem enforces the third axiom **A3** as long as all vectors involved are normalized; that is, has length (norm) one. This situation is depicted in Figure 1.

The situation is symmetric in a sense that reflects the duality between observable and state observed: Suppose now that the state |ψ〉 is “completed” by other vectors to form an entire context $C^{\prime }$. Then, one could consider this context $C^{\prime }$, including |ψ〉 to be“probe” vectors, now identified as states, in the original context C. Very similarly, probability measures adhering to Axioms **A1**–**A3** can be constructed by, say, for instance, $P_{E_{\psi }}\left(E_{i}\right)$

It is important to keep in mind that, although Gleason’s ansatz is about a single context C, it is valid for all contexts; indeed, formally, for a continuum of contexts represented by the continuum of possible orthonormal bases of *D*-dimensional Hilbert space. Every such context entails a particular view on the state |ψ〉; and there is a continuum of such views on the state |ψ〉.

Furthermore, there is a symmetry between the two contexts C and $C^{\prime }$ involved. We may call $C^{\prime }$ the “preparation context” and C the “measurement context,” but these denominations are purely conventional. In this sense, it is a matter of convention if we consider “C probing $C^{\prime }$” or “$C^{\prime }$ probing C.”

There is one “privileged view” on the preparation context $C^{\prime }$, that is the view obtained if both the preparation and measurement contexts coincide: $C=C^{\prime }$. Under such circumstances, the observables are value definite: their values coincide with those of the preparation.

## 3. Contexts in Partition Logics and Their Probabilities

This section is a reminder rather than an exposition [4,5,6,7,8,9,10] of partition logics. Suffice it to say that partition logics are probably the most elementary generalization of Boolean algebras: they are the Boolean subalgebras associated with sets of partitions of a given set, which are “pasted” or “stitched” together at their common elements; similar to contexts (blocks, subalgebras) in quantum logic. The main difference is that the latter is a continuous logic based on geometrical entities (vectors), whereas partition logics are discrete, finite algebraic structures based on sets of partitions of a given set. Nevertheless, for empirical purposes, it is always possible to come up with a partition logic mimicking the respective quantum logic [11]. Partition logics have two known model realization: automaton logics [12,13,14] and generalized urn models [15,16,17].

Just like classical probabilities on Boolean logics, the probabilities on Boolean structures are formed by a convex summation of all two-valued measures [9,10,18], corresponding to ball types. Such probabilities will henceforth be called (quasi)classical.

## 4. Probabilities on Pastings or Stitchings of Contexts

From dimension D≥3 onwards, contexts can be non-trivially connected or intertwined [1] in up to D−2 common elements. Such intertwining chains of contexts give rise to various apparently “non-classical” logics; and a wealth (some might say a plethora) of publications dealing with ever-increasing “strange” or “magic” properties of observables hitherto unheard of in classical physics. The following logics have a realization in (mostly three-dimensional if not stated otherwise) Hilbert space. For concrete parametrizations, the reader is either referred to the literature or to a recent survey (Chapter 12, [10]).

On such pastings of contexts, (quasi)classical probabilities and their bounds, termed conditions of possible experience by Boole (p. 229, [19]), can be obtained in three steps [8,9,10,18]:

(i) Enumerate all truth assignments (or two-{0,1}-valued measures or states) $v_{i}$.
(ii) The (quasi)classical probabilities are obtained by the formation of the convex sum $\sum_{i}\lambda_{i}v_{i}$ over all such states obtained in (i), with $0\leq \lambda_{i}\leq 1$ and $\sum_{i}\lambda_{i}=1$.
(ii) The Bell-type bounds on probabilities and expectations are obtained by bundling these truth assignments into vectors, one per two-valued measure, with the coordinates representing the respective values of those states on the atoms (propositions, observables) of the logic; and by subsequently solving the hull problem for a convex polytope whose vertices are identified with the vectors formed by all truth assignments [20,21,22,23].

In what follows, some such quantum logics will be enumerated whose quantum probabilities co-exist and sometimes violate their (quasi)classical probabilities, if they exist. Such violations can be expected to occur quite regularly, as (although in both cases, the probability Axioms **A1**–**A3** are satisfied for mutually-compatible observables) the quantum probabilities are formed very differently from the (quasi)classical ones; that is, not by convex sums as in the (quasi)classical case, but by scalar products among vectors.

### 4.1. Triangular and Square Logics in Four Dimensions

**Figure 2:**
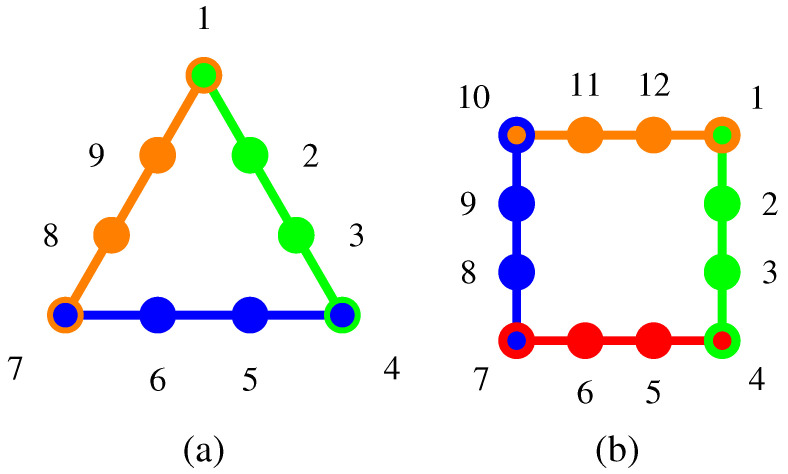
Informally, Greechie (or, in different wording, orthogonality) diagrams [24] represent contexts by smooth curves such as straight lines or circles. The atoms are represented by circles. Two intertwining contexts are represented by “broken” (not smooth), but connected lines. (**a**) Greechie orthogonality diagram of triangle logic in four dimensions, realized by (from the top) $1:\frac{1}{2}{\left(1,1,1,1\right)}^{⊺}$, $2:\frac{1}{\sqrt{2}}{\left(1,0,-1,0\right)}^{⊺}$, $3:\frac{1}{\sqrt{2}}{\left(0,1,0,-1\right)}^{⊺}$, $4:\frac{1}{2}{\left(-1,1,-1,1\right)}^{⊺}$, $5:\frac{1}{\sqrt{2}}{\left(0,1,1,0\right)}^{⊺}$, $6:\frac{1}{\sqrt{2}}{\left(1,0,0,1\right)}^{⊺}$, $7:\frac{1}{2}{\left(1,1,-1,-1\right)}^{⊺}$, $8:\frac{1}{\sqrt{2}}{\left(0,0,1,-1\right)}^{⊺}$ and $9:\frac{1}{\sqrt{2}}{\left(1,-1,0,0\right)}^{⊺}$. (**b**) Greechie orthogonality diagram of triangle logic in four dimensions, realized by (from the top right) $1:{\left(1,0,0,0\right)}^{⊺}$, $2:\frac{1}{\sqrt{2}}{\left(0,1,0,1\right)}^{⊺}$, $3:\frac{1}{\sqrt{2}}{\left(0,1,0,-1\right)}^{⊺}$, $4:{\left(0,0,1,0\right)}^{⊺}$, $5:\frac{1}{\sqrt{2}}{\left(1,1,0,0\right)}^{⊺}$, $6:\frac{1}{\sqrt{2}}{\left(1,-1,0,0\right)}^{⊺}$, $7:{\left(0,0,0,1\right)}^{⊺}$ and $8:\frac{1}{\sqrt{2}}{\left(1,0,1,0\right)}^{⊺}$, $9:\frac{1}{\sqrt{2}}{\left(1,0,-1,0\right)}^{⊺}$, $10:{\left(0,1,0,0\right)}^{⊺}$, $11:\frac{1}{\sqrt{2}}{\left(0,0,1,1\right)}^{⊺}$, $12:\frac{1}{\sqrt{2}}{\left(0,0,1,-1\right)}^{⊺}$. (Not all orthogonality relations are represented.) The associated (quasi)classical probabilities are obtained from a convex summation over all truth assignments, and listed in Appendix A and Appendix B.

For geometric and algebraic reasons, there is no cyclic pasting of three or four contexts in three dimensions, but in four dimensions, this is possible; as depicted in Figure 2. The (quasi)classical probabilities are enumerated in Appendix A and Appendix B.

Summation of the (quasi)classical probabilities on the intertwining atoms of the triangle logic yields $p_{1}+p_{4}+p_{7}=\lambda_{1}+\lambda_{2}+\lambda_{7}+\lambda_{12}+\lambda_{13}+\lambda_{14}\leq 1$. However, the axioms of probability theory are too restrictive to allow for quantum violations of these probabilities: after all, these adjacent vertices are mutually orthogonal and thus are in the same context (augmented with the fourth atom of that context). Other inequalities, such as $p_{1}+p_{2}=\lambda_{1}+\lambda_{2}\leq p_{5}+p_{6}=\left(\lambda_{1}+\lambda_{3}+\lambda_{4}+\lambda_{8}+\lambda_{9}\right)+\left(\lambda_{2}+\lambda_{5}+\lambda_{6}+\lambda_{10}+\lambda_{11}\right)$, compare vertices with the adjacent “inner” atoms; but again, due to the probability Axiom **A3**, the quantum probabilities must obey these inequalities, as well.

Komei Fukuda’s cddlib package [25] can be employed for a calculation of the hull problem, yielding all Bell-type inequalities associated with the convex polytope, the vertices of which are associated with the 14 or 34 truth assignments (two-valued measures) on the respective triangle and square logics. It turns out that all of them are expressions of Axioms **A1**–**A3**, which are mandatory also for the quantum probabilities within contexts.

### 4.2. Pentagon (Pentagram) Logic

**Figure 3:**
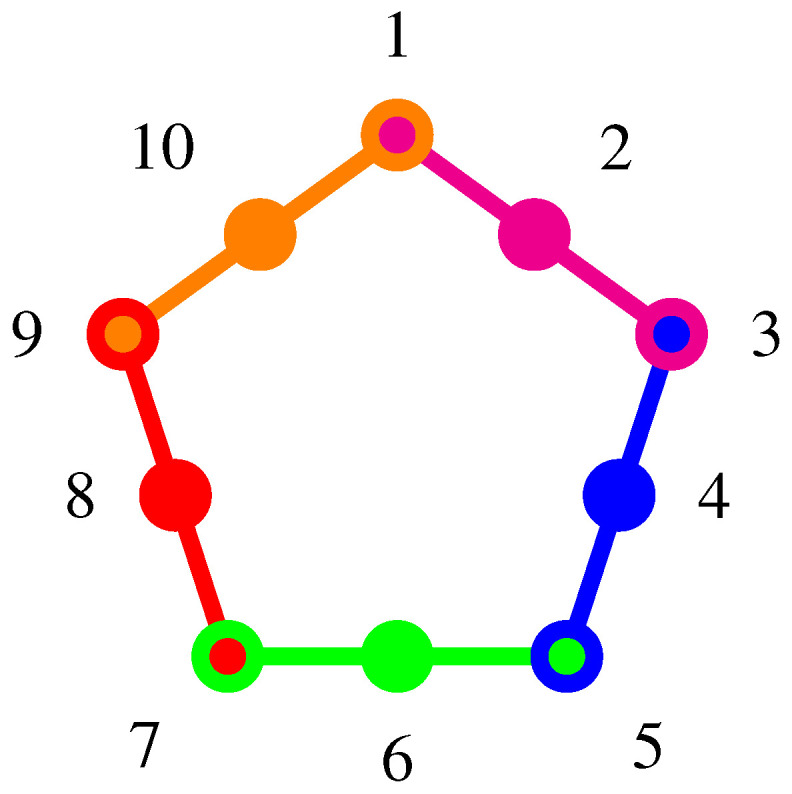
Greechie orthogonality diagram of the pentagon (pentagram) logic. The associated (quasi)classical probabilities are obtained from a convex summation over all truth assignments, and listed in Appendix C.

The pentagon (graph theoretically equivalent to a pentagram) logic is a cyclic stitching or pasting of five contexts [26,27,28,29,30,31,32], as depicted in Figure 3. The (quasi)classical probabilities (p. 289, Figure 11.8, [9]) can be obtained by taking the convex sum of all 11 two-valued measures [26], as listed in Appendix C. Because of the convex sum of all λ’s adds up to one, the sum of the (quasi)classical probabilities enumerated in Equation (A3), taken merely on the five intertwining observables, yields:

(3) $$\begin{array}{c} p_{1}+p_{3}+p_{5}+p_{7}+p_{9}\\ =\lambda_{1}+\lambda_{4}+\lambda_{7}+\lambda_{9}+\lambda_{10}+2\left(\lambda_{2}+\lambda_{3}+\lambda_{5}+\lambda_{6}+\lambda_{8}\right)\\ \leq 2\sum_{i=1}^{11}\lambda_{i}=2. \end{array}$$

This inequality is in violation of quantum predictions [30,32] of $\sqrt{5}>2$. Note that, in order to obtain the probabilities on the five intertwining observables (vertices), all of them need to be determined. However, only adjacent pairs share a common context. Therefore, at least three incompatible measurement types are necessary.

### 4.3. Specker Bug Logic with the True-Implies-False Property

**Figure 4:**
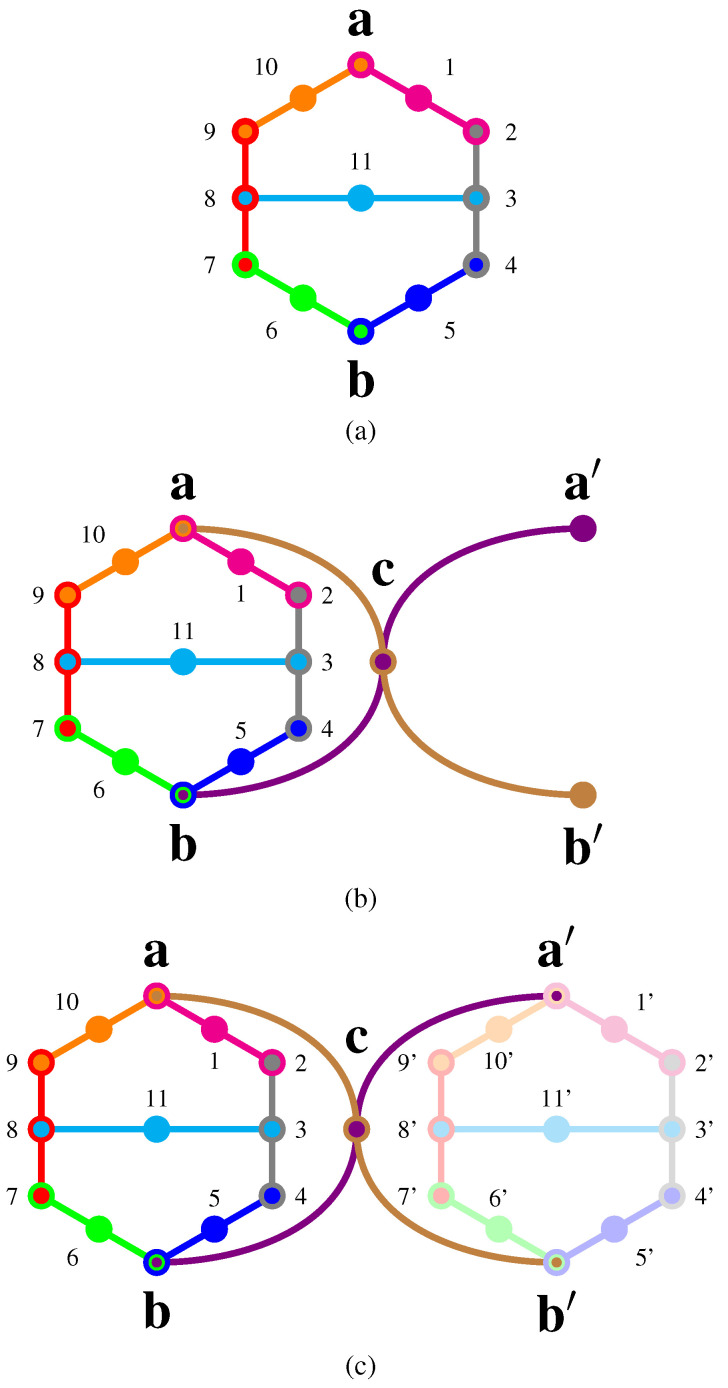
Greechie orthogonality diagram of (**a**) the Specker bug logic (Figure 1, p. 182, [33]). A proof that, if the system is prepared in state a, then classical (non-contextual) truth assignments require b not to occur proceeds as follows: In such a truth assignment, as per Axiom **A3**, there is only one true atom per context; all the others have to be false. In a proof by contradiction, suppose that both a and b are true. Then, all atoms connected to them (2,4,7,9) must be false. This in turn requires that the observables (3,8) connecting them must both be true. Alas, those two observables (3,8) are connected by a “middle” context {3,11,8} . But the occurrence of two true observables within the same context is forbidden by Axiom **A3**. The only consistent alternative is to disallow b to be true if a is assumed to be true; or conversely, to disallow a to be true if b is assumed to be true. (**b**) Greechie orthogonality diagram of a Specker bug logic extended by two contexts, which has the true-implies-true property on $a^{\prime }$, given a to be true ($\Gamma_{1}$, p. 68, [34]). (**c**) Greechie orthogonality diagram of a combo of two Specker bug logics ($\Gamma_{3}$, p. 70, [34]). If a is assumed to be true, then the remaining atoms in the context $\left\{a,c,b^{\prime }\right\}$ connecting a with $b^{\prime }$ and, in particular, c have to be false. Furthermore, if a is true, then b is false. Therefore, $a^{\prime }$ needs to be true if b and c both are false, because they form the context $\left\{b,c,a^{\prime }\right\}$. This argument is valid even in the absence of a second Specker bug logic. Introduction of a second Specker bug logic ensures the converse: whenever $a^{\prime }$ is true, a must be true, as well. Therefore, a and $a^{\prime }$ (and by symmetry, also b and $b^{\prime }$) cannot be separated by any truth assignment.

A pasting of two pentagon logics, the “Specker bug” logic, has been introduced (Figure 1, p. 182, [33]) and used ($\Gamma_{1}$, p. 68, [34]) by Kochen and Specker and discussed by many researchers [35,36,37]; see also (Figure B.l, p. 64, [38]), (pp. 588–589, [39]), (Section IV, Figure 2, [40]) and (p. 39, Figure 2.4.6, [41]). It is a pasting [27,42] of seven contexts in such a tight way (cf. Figure 4a) that preparation of a (quasi)classical system in state a entails the non-occurrence of observable b. As has been observed by Stairs (pp. 588–589, [39]) and Clifton (Sections II and III, Figure 1, [40,43,44]), this is no longer the case for quantum states and quantum observables. Therefore, if one prepares a system in a state |a〉 and measures $E_{b}=\left|b\rangle \langle b\right|$, associated with state |b〉, then the mere occurrence of |b〉 implies the non-classicality of the quantized system.

Again, the (quasi)classical probabilities (p. 286, Figure 11.5(iii), [9]) enumerated in Appendix D can be obtained by taking the convex sum of all 14 two-valued measures (p. 579, Table 7, [8]). Pták and Pulmannová (p. 39, Figure 2.4.6, [41]), as well as Pitowsky in (p. 402, Figure 2, [36]) and (pp. 224, 225, Figure 10.2, [37]) noted that, for (quasi)classical probabilities, including ones on partition logics, the sum of the probabilities on |a〉 and |b〉 must not exceed $\frac{3}{2}$. Therefore, both cannot be true at the same time, because this would result in their sum being two. This might be called a true-implies-false property [45] (also known as the one-zero rule [46]) on the atoms a and b.

Actually, this classical bound can be tightened by explicity summing the (quasi)classical probabilities of a and b enumerated in Equation (A5). Because of the convex sum of all λ’s adds up to one, this yields:

(4) $$p_{a}+p_{b}=\lambda_{1}+\lambda_{2}+\lambda_{3}+\lambda_{6}+\lambda_{13}+\lambda_{14}\leq \sum_{i=1}^{14}\lambda_{i}=1.$$

This inequality is in violation of quantum predictions for a system prepared in state |a〉; in this case [47], $\frac{10}{9}>1$.

Indeed, Cabello [47] (see also his dissertation (pp. 55–56, [48])) pointed out that in three dimensions, |a〉 and |b〉 must be at least an angle $∠\left(a,b\right)\geq \mathrm{arcsec}\left(3\right)=\arccos\left(\frac{1}{3}\right)=\frac{\pi }{2}-\mathrm{arccot}\left(2\sqrt{2}\right)=\arctan\left(2\sqrt{2}\right)$ apart. Therefore, the probability of finding a state prepared along $|a\rangle \equiv {\left(\begin{array}{c} 1,0,0 \end{array}\right)}^{⊺}$ in a state $|b\rangle \equiv {\left(\begin{array}{c} \cos∠(a,b),\sin∠(a,b),0 \end{array}\right)}^{⊺}$ cannot exceed ${\left|\langle b|a\rangle \right|}^{2}=1/9$. Thus, in at most one-ninth of all cases will quantum mechanical probabilities violate the classical ones, as the classical prediction demands zero probability to measure b, given a (this prediction is relative to the assumption of non-contextuality, such that the truth assignment is independent of the particular context). For a concrete “optimal” realization (p. 206, Figure 1, [49]) (see also (Figure 4, p. 5387, [50])), take $|a\rangle =\frac{1}{\sqrt{3}}{\left(\begin{array}{c} 1,\sqrt{2},0 \end{array}\right)}^{⊺}$ and $|b\rangle =\frac{1}{\sqrt{3}}{\left(\begin{array}{c} -1,\sqrt{2},0 \end{array}\right)}^{⊺}$, which yield $\left|\langle b|a\rangle \right|=\frac{1}{3}$.

**Figure 5:**
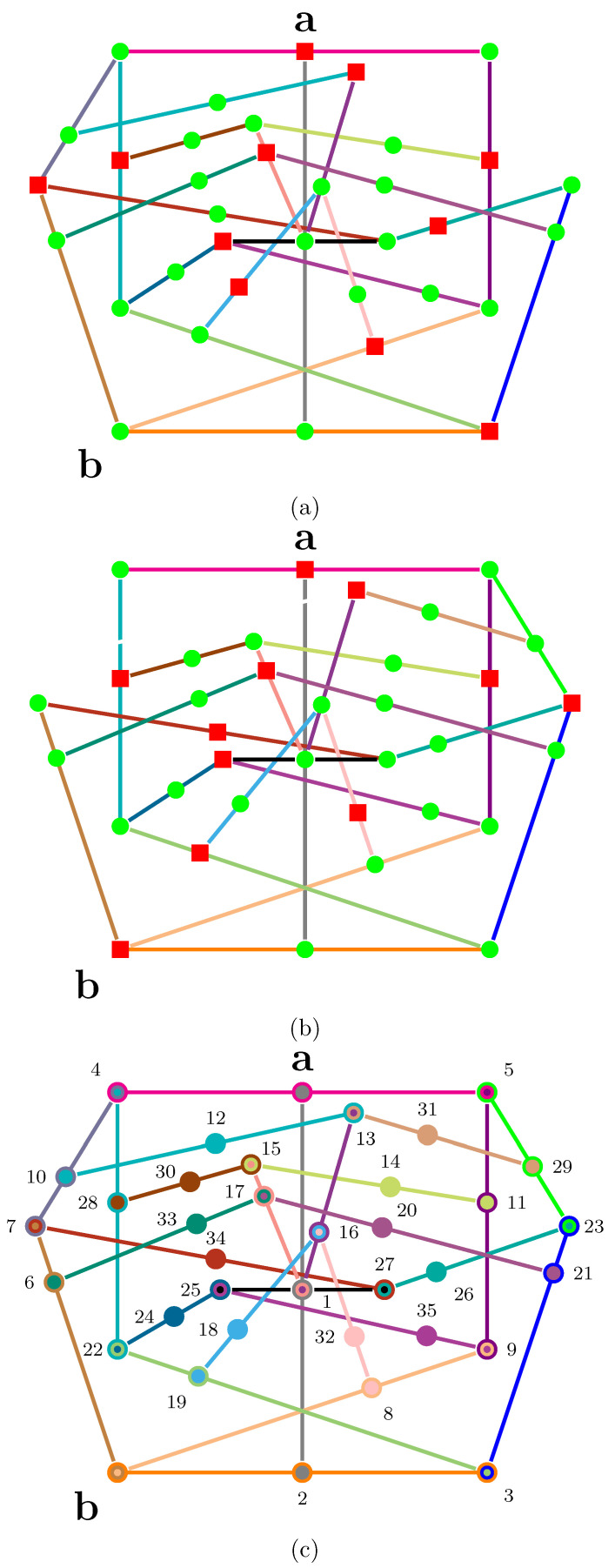
Greechie orthogonality diagram of a logic (Figure 2, p. 102201-8, [51]) realizable in $R^{3}$ (**a**) with the true-implies-false property, (**b**) with the true-implies-true property and (**c**) with the true-implies-value indefiniteness (neither true nor false) property on the atoms a and b, respectively. (a,b) contain the single (out of 13) value assignment that is possible and for which a is true. All eight value assignments of the logic depicted in (c) require a to be false.

Another true-implies-false configuration depicted in Figure 5a has an immediate quantum realization (Table 1, p. 102201-7, [51]) for ${\left|\langle a|b\rangle \right|}^{2}=\frac{1}{2}$ and can be constructively (i.e., algorithmically computable) extended to arbitrary angles between non-collinear and non-orthogonal vectors.

### 4.4. Combo of Specker Bug Logic with the True-Implies-True, as Well as Inseparability Properties

This non-classical behavior can be “boosted” by an extension of the Specker bug logic ($\Gamma_{1}$, p. 68, [34]), including two additional contexts $\left\{a,c,b^{\prime }\right\}$, as well as $\left\{b,c,a^{\prime }\right\}$, as depicted in Figure 4b. It implements a true-implies-true property [45] (also known as the one-one rule [46]) for a and $a^{\prime }$. Cabello’s bound on the angle ∠(a,b) between a and b mentioned earlier results in bounds between a and $a^{\prime }$, as well as b and $b^{\prime }$: since a and $b^{\prime }$, as well as b and $a^{\prime }$ are orthogonal, that is, $∠\left(a,b^{\prime }\right)=∠\left(b,a^{\prime }\right)=\frac{\pi }{2}$, it follows for planar configurations that $∠\left(a,a^{\prime }\right)=∠\left(b,a^{\prime }\right)-∠\left(a,b\right)\leq \frac{\pi }{2}-\arccos\left(\frac{1}{3}\right)=\mathrm{arccot}\left(2\sqrt{2}\right)=\mathrm{arccsc}\left(3\right)=\arcsin\left(\frac{1}{3}\right)$. For symmetry reasons, the same estimate holds for planar configurations between b and $b^{\prime }$. For non-planar configurations, the angles must be even less than for planar ones.

True-implies-true properties have also been studied by Stairs (pp. 588–589, note added in proof, [39]); Clifton (Sections II and III, Figure 1, [40,43,44]) presents a similar argument, based on another true-implies-true logic inspired by Bell (Figure C.l, p. 67, [38]) (cf. also Pitowsky (p. 394, [52])), on the Specker bug logic (Section IV, Figure 2, [40]). More recently, Hardy [53,54,55], as well as Cabello and García-Alcaine and others [32,56,57,58,59,60] have discussed such scenarios.

Another true-implies-true configuration depicted in Figure 5b has an immediate quantum realization (Table 1, p. 102201-7, [51]) for ${\left|\langle a|b\rangle \right|}^{2}=\frac{1}{2}$ and can be extended to arbitrary angles between non-collinear and non-orthogonal vectors.

A combo of Specker bug logics renders a non-separable set of two-valued states ($\Gamma_{3}$, p. 70, [34]): in the logic depicted in Figure 4c, a and $a^{\prime }$, as well as b and $b^{\prime }$ cannot be “separated” from one another by any non-contextual (quasi)classical truth assignment enumerated in Appendix D. Kochen and Specker (Theorem 0, p. 67, [34]) pointed out that this type of inseparability is a necessary and sufficient condition for a logic to be not embeddable in any classical Boolean algebra. Therefore, whereas both the Specker bug logic, as well as its extension true-implies-true logic can be represented by a partition logic, the combo Specker bug logic cannot.

### 4.5. Logics Inducing Partial Value (In)Definiteness

Probably the strongest forms of value indefiniteness [61,62] are theorems [51,63,64] stating that relative to reasonable (admissibility, non-contextuality) assumptions, if a quantized system is prepared in some pure state |a〉, then any observable that is not identical or orthogonal to |a〉 is undefined. That is, there exist finite systems of quantum contexts whose pastings are demanding that any pure state |b〉 not belonging to some context with |a〉 can neither be true, nor false; else a complete contradiction would follow from the assumption of classically pre-existent truth values on some pasting of contexts such as the Specker bug logic.

What does “strong” mean here? Suppose one prepares the system in a particular context C such that a single vector |a〉∈C is true; that is, |a〉 has probability measure of one when measured along C. Then, if one measures a complementary variable |b〉 and |b〉 is sufficiently separated from |a〉 (more precisely, at least an angle $\arccos\left(\frac{1}{3}\right)$ apart for the Specker bug logic), then intertwined quantum propositional structures (such as the Specker bug logic) exist, which, interpreted (quasi)classically, demand that |b〉 can never occur (cannot be true); and yet, quantum systems allow |b〉 to occur. Likewise, other intertwined contexts that correspond to true-implies-true configurations of quantum observables (termed Hardy-like [53,54,55] by Cabello [60]) (quasi)classically imply that some endpoint $|b^{\prime }\rangle$ must always occur, given |a〉 is true. Yet, quantum mechanically, since |a〉 and $|b^{\prime }\rangle$ are not collinear, quantum mechanics predicts that occasionally, $|b^{\prime }\rangle$ does not occur. In the “strongest” form [51,63,64] of classical “do’s and don’ts”, there are no possibilities whatsoever for an observable proposition to be either true or false. That is, even if the Specker bug simultaneously allows some |a〉 to be true and |b〉 to be false (although disallowing the latter to be true), there is another, supposedly more sophisticated finite configuration of intertwined quantum contexts, that can be constructively enumerated and that disallows |b〉 even to be false (it cannot be true either).

For the sake of an explicit example, take the logic (Figure 2, p. 102201-8, [51]) depicted in Figure 5c. It is the composite of two logics depicted in Figure 5a,b, which perform very differently at b given a to be true: whereas (a) implements a true-implies-false property, (b) has a true-implies-true property for the atoms a and b, respectively. Both (a) and (b) are proper subsets (lacking two contexts) of the logic in Figure 5c; and apart from their difference in four contexts, are identical.

More precisely, as explicated in Appendix E, both of these logics (a) and (b) allow 13 truth assignments (two-valued states), but only a single one allows a to be true on either of them (this uniqueness is not essential to the argument). The logic in (c) allows for eight truth assignments, but all of them assign falsity to a. By combining the logics (a) and (b), one obtains (c) which, if a is assumed to be true, implies that b can neither be true (this would contradict the true-implies-false property of (a)) nor can it be false, because this would contradict the true-implies-true property of (b). Hence, we are left with the only consistent alternative (relative to the assumptions): that a system prepared in state a must be value indefinite for observable b. Thereby, as the truth assignment on b is not defined, it must be partial on the entire logic depicted in Figure 5c.

The scheme of the proof is as follows:

(i) Find a logic (collection of intertwined contexts of observables) exhibiting a true-implies-false property on the two atoms a and b.
(ii) Find another logic exhibiting a true-implies-true property on the same two atoms a and b.
(iii) Then, join (paste) these logics into a larger logic, which, given a, neither allows b to be true nor false. Consequently, b must be value indefinite.

**Figure 6:**
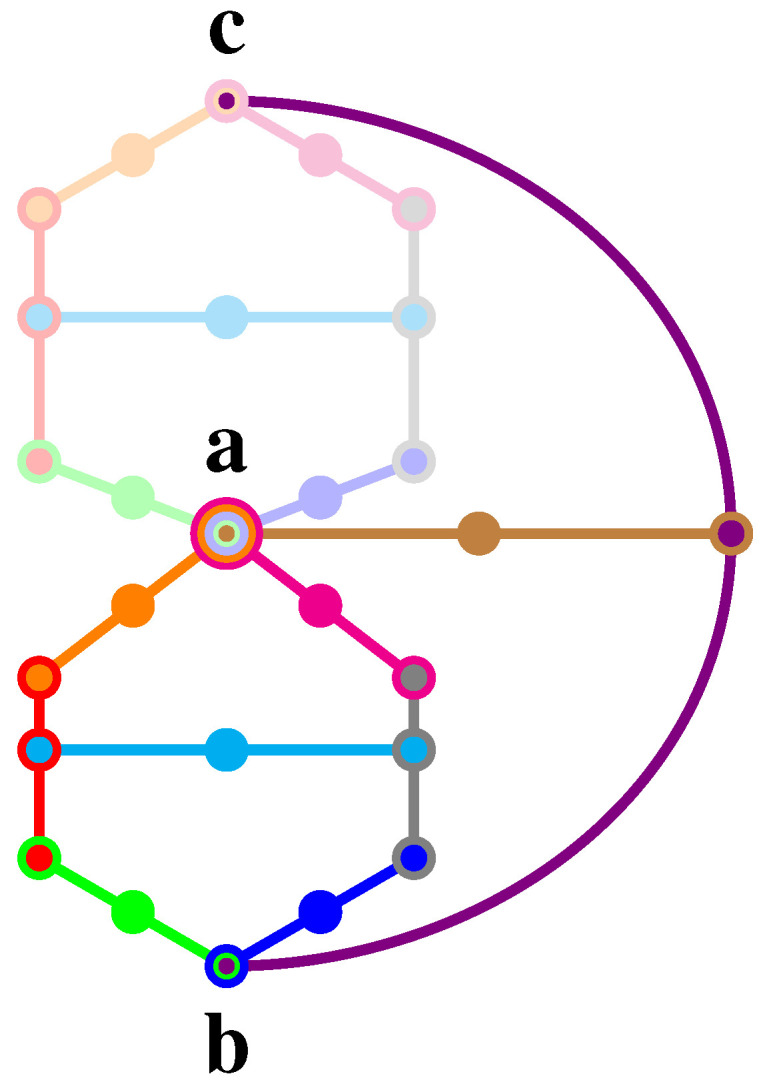
Greechie orthogonality diagram of a logic that is value indefinite on b (as well as on c for symmetry reasons), given a is true; alas, such a logic has no realization in three-dimensional Hilbert space, as the angles ∠(a,b) between a and b should simultaneously obey 1.2≈arcsec(3)≤∠(a,b)≤arccsc(3)≈0.3.

The most suggestive candidate for such a pasting is, however, unavailable: it is the combination of a Specker bug logic and another, extended Specker bug logic, as depicted in Figure 6. Such a logic cannot be realized in three dimensions, as the angles cannot be chosen consistently; that is, obeying the Cabello bounds on the relative angles, respectively.

The latter result about the partiality of the truth assignment has already been discussed by Pitowsky [61], and later by Hrushovski and Pitowsky [62]. It should also be mentioned that the logic (c) has been realized with a particular configuration in three-dimensional real Hilbert space (Tables I and II, p. 102201-7, [51]), which are an angle $∠\left(a,b\right)=\arccos\left(\frac{1}{\sqrt{2}}\right)$ apart, but as has been mentioned earlier, this kind of value indefiniteness on any particular state b, given that the system has been prepared in state a, can be constructively obtained by an extension of the above configuration whenever a and b are neither collinear (in this case, b would be true) nor orthogonal (in this case, b would be false). Therefore, basically, all states not identical (or orthogonal) to the state prepared must be value indefinite.

All three logics in Figure 6a–c have another non-classical feature: they are non-unital [49], meaning that the truth assignments on some of their atoms can only acquire the value as false, regardless of the preparation. That is, in this “state-independent” form, whenever a proposition corresponding to such an atom is measured to be true, this can be interpreted as the indication of non-classicality (note that one can always rotate the entire set of rays so that this particular atom coincides with some observable measured.).

## 5. Propositional Logic Does Not Uniquely Determine Probabilities

By now, it should be clear that the propositional structure does in general not uniquely determine its probabilities. The Specker bug in Figure 4a serves as a good example of that: it supports (quasi)classical probabilities, explicitly enumerated in(p. 286, Figure 11.5(iii), [9]) and (p. 91, Figure 12.10, [10]), which are formed by convex combinations of all two-valued states on them.

Other propositional structures such as the pentagon logic support “exotic” probability measures [26], which do not vanish at their interlink observables and are equally weighted with value $\frac{1}{2}$ there. This measure is neither realized in the (quasi)classical partition logic setup explicitly discussed in (p. 289, Figure 11.8, [9]) and (p. 88, Figure 12.8, [10]), nor in quantum mechanics. It remains to be seen if a more general theory of probability measures based on Axioms **A1**–**A3** can be found.

## 6. Some Platonist Afterthoughts

The author’s not-so-humble reading of all these aforementioned “mind-boggling” non-classical quantum predictions is a rather sober one: in view of the numerous indications that classical value definiteness cannot be extended to more than a single context, the most plausible supposition is that, besides exotic possibilities [65,66], ontologically, there is only one such “Realding” (indeed, a rather obvious candidate suggesting itself as ontology): a single vector, or rather a single context. Quantized systems can be completely and exhaustively characterized by a unique context and a “true” proposition within this context.

Suppose for a moment that this hypothesis is correct and that there is no ontology, no “Realding,” beyond a single context. There is one preferred view, namely the context identical to the context in which the system has been prepared, and all but one epistemic view.

Yet, a confusing experience is the apparent ease with which an experimenter appears to measure, without any difficulty, a context or (maximal) observable not (or only partly through intertwines) matching the preparation context. In such a situation, one may assume that the measurement grants an “imperfect” view on the preparation context. In this process, information, in particular the relative locatedness of the measurement context with respect to the preparation context, is augmented by properties of the measurement device, thereby effectively generating entanglement [67,68] via context translation [69]. Frames of reference that do not coincide with the “Realding” or preparation context necessarily include stochastic elements that are not caused or determined by any property of the formerly individual “Realding.” One may conclude [70] with Bohr’s 1972 Como lecture (p. 580, [71]) that “any observation of atomic phenomena will involve an interaction with the agency of observation not to be neglected. Accordingly, an independent reality in the ordinary physical sense can neither be ascribed to the phenomena nor to the agencies of observation.” That is, any interaction between the previously separated individual object and the measurement device results in a joint physical state that is no longer determined by the states of the (previously) individual constituents [68,72]. Instead, the joint state exhibits what Schrödinger later called entanglement [67]. Entanglement is characterized by a value definite relational [73] or collective (re-)encoding of information with respect to the constituent parts, thereby (since the unitary quantum evolution is injective) resulting in the value indefiniteness of the previously individual and separate parts. As a result, knowledge about observations obtained by different contexts than the preparation context are necessarily (at least partially in the sense of the augmented information from the measurement device) epistemic.

Another possible source of perplexity might be the various types of algebraic or logical structures involved. Classically, empirical logics are Boolean algebras. Then, in a first step towards non-classicality, there are partition logics that are not Boolean any longer (they feature complementarity through non-distributivity), but nevertheless still allow for a certain type of (quasi)classicality; that is, a separating and unital set of two-valued states. Then, further on this road, there are (finite) quantum logics that do not allow any definite state at all.

One might be puzzled by the fact that there exist “intermediate” logics, such as the Specker bug or the pentagon (pentagram) logic discussed in Section 4.2 and Section 4.3 that still allow (even classical) simultaneous value indefiniteness, although they contain observables that are mutually complementary (non-collinear and non-orthogonal). However, this apparent paradox should rather be interpreted epistemically, as means (configuration) relative [74]: in the case of the pentagon, we have decided to concentrate on 10 observables in a cyclic pasting of five contexts, but we have thereby implicitly chosen to “look the other way” and disregard the abundance of other observables that impose much more stringent conditions on the value definiteness of the observables in the pentagon logic than the pentagon logic itself.

Therefore, properties such as the true-implies-false, the true-implies-true properties, as well as inseparability and even value indefiniteness are means relative and valid only if one restricts or broadens one’s attention to sometimes very specific, limited sublogics of the realm of all conceivable quantum logics, which are structures formed by perpendicular projection operators in Hilbert spaces of dimension larger than two.

Pointedly stated, sets of intertwining contexts connecting two (or more) relevant complementary observables a and b should be considered as totally arbitrary when it comes to the inclusion or exclusion of particular contexts interconnecting them: there is neither a necessity nor even a compelling reason to take into account one such structure and disregard another, or favor one over the other. Indeed, in an extreme, sui generis form of the argument, suppose a single quantum is prepared in some state a. Then, every single outcome of a measurement of every complementary (non-collinear and non-orthogonal relative to the state prepared) quantum observable may be considered as “proof” or “certification of non-classicality” (or, in different terminology, “contextuality”). Those observable can be identified with the “endpoint” b of either some true-implies-false, or alternatively true-implies-true configuration (say the one sketched in Figure 6a,b), depending on whether the classical false or true predictions need to contradict the particular outcome, respectively. For quantum logics with a unital set of two-valued states, such as the logics depicted by Tkadlec (p. 207, Figure 2, [49]) or the ones in Figure 6a,b, one could even get rid of the state preparation if b occurs and is identified with an observable that, according to the classical predictions associated with that logic, cannot occur. There is no principle that could prevent us from arguing that way if we insist on the simultaneous existence of multiple contexts encountered in quantum mechanics. Indeed, are not intertwining contexts scholastic [75] sophisms in desperate need of deconstruction?

An interesting historical question arises: Kochen and Specker, in a succession of papers on partial algebras [33,34,76], have insisted that logical operations should only be defined within contexts and must not be applied to propositions outside of it. Yet, they have considered extended counterfactual structures of pasted context, ending up in a holistic argument involving complementary observables. Of course, an immediate reply might be that without intertwined contexts, there cannot be any non-trivial (non-classical, non-Boolean) configuration of observables that is of any interest.

For the reasons mentioned earlier, the emphasis should not be on “completing” quantum mechanics by some sort of hidden parameter theory, such as, for instance, Valentini [77] envisioning a theory that is to quantum mechanics as statistical physics is to thermodynamics, but just the opposite: the challenge is to acknowledge the scarcity of resources, the “Realding” or physical state as a mere vector, despite the continuum of possible views on it, resulting in an illusory over-abundance and over-determination.

In this line of thought, the question of what might be the reason behind the futility to co-define non-commuting quantum observables (from two or more different contexts) simultaneously should be answered in terms of a serious lack of a proper perspective of what one is dealing with: metaphorically speaking, it is almost as if one pretends to take a $360^{\circ }$ panorama of what lies in the outside world, while actually merely taking photos from some sort of echo chamber, or house of mirrors, partly reflecting what is in it, and partly reproducing the observer (photographer) in almost endless reflections. Stitching together photos from these reflections yields a panorama of one and the same object in seemingly endless varieties. In this way, one might end up with a horribly distorted image of this situation; and with the inside turned outside.

This is not dissimilar to what Plato outlined in the Republic’s cave metaphor (Book 7, 515c, p. 221, [78]): “what people in this situation would take for truth would be nothing more than the shadows of the manufactured objects.” In the quantum transcription of this metaphor, the vectors are the objects, and the shadows taken for truth are the views on these objects, mediated or translated [69] by arbitrary mismatching contexts.

## Appendix A. Two-Valued States, (Quasi)Classical Probabilities on the Triangular Logic in Four Dimensions

The two-valued states (also known as truth tables) have been enumerated by Josef Tkadlec’s Pascal program 2states [79]. Implicitly, the convex sums over the respective probabilities encode the truth tables, as, on any particular atom, the *i*’th truth table entry is one if $\lambda_{i}$ appears in the listing of the classical probability $p_{i}$. Otherwise, the *i*’th truth table entry is zero.

The bounds for classical probabilities have been obtained by Komei Fukuda’s cddlib package [25].

There are nine propositions forming three contexts {1,2,3,4}, {4,5,6,7} and {7,8,9,1} allowing 14 (separating, unital) two-valued states whose convex sum yields the following (quasi)classical probabilities:

(A1) $$\begin{array}{cc} p_{1}= & \lambda_{1}+\lambda_{2},\\ p_{2}= & \lambda_{3}+\lambda_{4}+\lambda_{5}+\lambda_{6}+\lambda_{7},\\ p_{3}= & \lambda_{8}+\lambda_{9}+\lambda_{10}+\lambda_{11}+\lambda_{12},\\ p_{4}= & \lambda_{13}+\lambda_{14},\\ p_{5}= & \lambda_{1}+\lambda_{3}+\lambda_{4}+\lambda_{8}+\lambda_{9},\\ p_{6}= & \lambda_{2}+\lambda_{5}+\lambda_{6}+\lambda_{10}+\lambda_{11},\\ p_{7}= & \lambda_{7}+\lambda_{12},\\ p_{8}= & \lambda_{3}+\lambda_{5}+\lambda_{8}+\lambda_{10}+\lambda_{13},\\ p_{9}= & \lambda_{4}+\lambda_{6}+\lambda_{9}+\lambda_{11}+\lambda_{14}. \end{array}$$

## Appendix B. Truth Assignments, (Quasi)Classical Probabilities on the Square Logic in Four Dimensions

There are 12 propositions forming four contexts {1,2,3,4}, {4,5,6,7}, {7,8,9,10} and {10,11,12,1} allowing 34 (separating, unital) two-valued states whose convex sum yields the following (quasi)classical probabilities:

(A2) $$\begin{array}{cc} p_{1}= & \lambda_{1}+\lambda_{2}+\lambda_{3}+\lambda_{4}+\lambda_{5},\\ p_{2}= & \lambda_{6}+\lambda_{7}+\lambda_{8}+\lambda_{9}+\lambda_{10}+\lambda_{11}\\ & +\lambda_{12}+\lambda_{13}+\lambda_{14}+\lambda_{15}+\lambda_{16}+\lambda_{17},\\ p_{3}= & \lambda_{18}+\lambda_{19}+\lambda_{20}+\lambda_{21}+\lambda_{22}+\lambda_{23}\\ & +\lambda_{24}+\lambda_{25}+\lambda_{26}+\lambda_{24}+\lambda_{28}+\lambda_{29},\\ p_{4}= & \lambda_{30}+\lambda_{31}+\lambda_{32}+\lambda_{33}+\lambda_{34},\\ p_{5}= & \lambda_{1}+\lambda_{2}+\lambda_{6}+\lambda_{7}+\lambda_{8}+\lambda_{9}\\ & +\lambda_{10}+\lambda_{18}+\lambda_{19}+\lambda_{20}+\lambda_{21}+\lambda_{22},\\ p_{6}= & \lambda_{3}+\lambda_{4}+\lambda_{11}+\lambda_{12}+\lambda_{13}+\lambda_{14}\\ & +\lambda_{15}+\lambda_{23}+\lambda_{24}+\lambda_{25}+\lambda_{26}+\lambda_{27},\\ p_{7}= & \lambda_{5}+\lambda_{16}+\lambda_{17}+\lambda_{28}+\lambda_{29},\\ p_{8}= & \lambda_{1}+\lambda_{3}+\lambda_{6}+\lambda_{7}+\lambda_{11}+\lambda_{12}\\ & +\lambda_{18}+\lambda_{19}+\lambda_{23}+\lambda_{24}+\lambda_{30}+\lambda_{31},\\ p_{9}= & \lambda_{2}+\lambda_{4}+\lambda_{8}+\lambda_{9}+\lambda_{13}+\lambda_{14}\\ & +\lambda_{20}+\lambda_{21}+\lambda_{25}+\lambda_{26}+\lambda_{32}+\lambda_{33},\\ p_{10}= & \lambda_{10}+\lambda_{15}+\lambda_{22}+\lambda_{27}+\lambda_{34},\\ p_{11}= & \lambda_{6}+\lambda_{8}+\lambda_{11}+\lambda_{13}+\lambda_{16}+\lambda_{18}\\ & +\lambda_{20}+\lambda_{23}+\lambda_{25}+\lambda_{28}+\lambda_{30}+\lambda_{32},\\ p_{12}= & \lambda_{7}+\lambda_{9}+\lambda_{12}+\lambda_{14}+\lambda_{17}+\lambda_{19}\\ & +\lambda_{21}+\lambda_{24}+\lambda_{26}+\lambda_{29}+\lambda_{31}+\lambda_{33}. \end{array}$$

## Appendix C. Two-Valued States, (Quasi)Classical Probabilities on the Pentagon (Pentagram) Logic in Three Dimensions

There are five contexts {1,2,3}, {3,4,5}, {5,6,7}, {7,8,9} and {9,10,1} allowing 11 (separating, unital) two-valued states [26] whose convex sum yields the following (quasi)classical probabilities:

(A3) $$\begin{array}{cc} p_{1}= & \lambda_{1}+\lambda_{2}+\lambda_{3},\\ p_{2}= & \lambda_{4}+\lambda_{5}+\lambda_{7}+\lambda_{9}+\lambda_{11},\\ p_{3}= & \lambda_{6}+\lambda_{8}+\lambda_{10},\\ p_{4}= & \lambda_{1}+\lambda_{2}+\lambda_{4}+\lambda_{7}+\lambda_{11},\\ p_{5}= & \lambda_{3}+\lambda_{5}+\lambda_{9},\\ p_{6}= & \lambda_{1}+\lambda_{4}+\lambda_{6}+\lambda_{10}+\lambda_{11},\\ p_{7}= & \lambda_{2}+\lambda_{7}+\lambda_{8},\\ p_{8}= & \lambda_{1}+\lambda_{3}+\lambda_{9}+\lambda_{10}+\lambda_{11},\\ p_{9}= & \lambda_{4}+\lambda_{5}+\lambda_{6},\\ p_{10}= & \lambda_{7}+\lambda_{8}+\lambda_{9}+\lambda_{10}+\lambda_{11}. \end{array}$$

## Appendix D. Truth Assignments, (Quasi)Classical Probabilities on the Specker Bug Combo Logic

The logic depicted in Figure 4c contains 27 propositions forming 16 contexts {a,1,2}, {2,3,4}, {4,5,b}, {b,6,7}, {7,8,9}, {9,10,a}, {3,8,11}, $\left\{a,c,b^{\prime }\right\}$, $\left\{b,c,a^{\prime }\right\}$, $\left\{a^{\prime },1^{\prime },2^{\prime }\right\}$, $\left\{2^{\prime },3^{\prime },4^{\prime }\right\}$, $\left\{4^{\prime },5^{\prime },b^{\prime }\right\}$, $\left\{b^{\prime },6^{\prime },7^{\prime }\right\}$, $\left\{7^{\prime },8^{\prime },9^{\prime }\right\}$, $\left\{9^{\prime },10^{\prime },a^{\prime }\right\}$ and $\left\{3^{\prime },8^{\prime },11^{\prime }\right\}$, allowing 82 non-separating on a/$a^{\prime }$ and b/$b^{\prime }$, unital two-valued states (not enumerated here because of volume). Nine and nine of these permit a, as well as $a^{\prime }$ and b, as well as $b^{\prime }$ to be true, respectively.

The logic depicted in Figure 4b contains 16 propositions forming nine contexts {a,1,2}, {2,3,4}, {4,5,b}, {b,6,7}, {7,8,9}, {9,10,a}, {3,8,11}, $\left\{a,c,b^{\prime }\right\}$ and $\left\{b,c,a^{\prime }\right\}$, allowing 22 (separating and unital) two-valued states, which, through their convex summation, yield the (quasi-)classical probabilities:

(A4) $$\begin{array}{cc} p_{a}= & \lambda_{1}+\lambda_{2}+\lambda_{3},\\ p_{b}= & \lambda_{8}+\lambda_{21}+\lambda_{22},\\ p_{a^{\prime }}= & \lambda_{1}+\lambda_{2}+\lambda_{3}+\lambda_{5}+\lambda_{7}+\lambda_{10}\\ & +\lambda_{12}+\lambda_{14}+\lambda_{16}+\lambda_{18}+\lambda_{20},\\ p_{b^{\prime }}= & \lambda_{5}+\lambda_{7}+\lambda_{8}+\lambda_{10}+\lambda_{12}+\lambda_{14}\\ & +\lambda_{16}+\lambda_{18}+\lambda_{20}+\lambda_{21}+\lambda_{22},\\ p_{c}= & \lambda_{4}+\lambda_{6}+\lambda_{9}+\lambda_{11}+\lambda_{13}+\lambda_{15}+\lambda_{17}+\lambda_{19},\\ p_{1}= & \lambda_{4}+\lambda_{5}+\lambda_{6}+\lambda_{7}+\lambda_{8}+\lambda_{9}\\ & +\lambda_{10}+\lambda_{11}+\lambda_{12}+\lambda_{13}+\lambda_{14},\\ p_{2}= & \lambda_{15}+\lambda_{16}+\lambda_{17}+\lambda_{18}+\lambda_{19}+\lambda_{20}+\lambda_{21}+\lambda_{22},\\ p_{3}= & \lambda_{1}+\lambda_{4}+\lambda_{5}+\lambda_{6}+\lambda_{7}+\lambda_{8},\\ p_{4}= & \lambda_{2}+\lambda_{3}+\lambda_{9}+\lambda_{10}+\lambda_{11}+\lambda_{12}+\lambda_{13}+\lambda_{14},\\ p_{5}= & \lambda_{1}+\lambda_{4}+\lambda_{5}+\lambda_{6}+\lambda_{7}+\lambda_{15}\\ & +\lambda_{16}+\lambda_{17}+\lambda_{18}+\lambda_{19}+\lambda_{20},\\ p_{6}= & \lambda_{2}+\lambda_{4}+\lambda_{5}+\lambda_{9}+\lambda_{10}+\lambda_{11}\\ & +\lambda_{12}+\lambda_{15}+\lambda_{16}+\lambda_{17}+\lambda_{18},\\ p_{7}= & \lambda_{1}+\lambda_{3}+\lambda_{6}+\lambda_{7}+\lambda_{13}+\lambda_{14}+\lambda_{19}+\lambda_{20},\\ p_{8}= & \lambda_{2}+\lambda_{9}+\lambda_{10}+\lambda_{15}+\lambda_{16}+\lambda_{21},\\ p_{9}= & \lambda_{4}+\lambda_{5}+\lambda_{8}+\lambda_{11}+\lambda_{12}+\lambda_{17}+\lambda_{18}+\lambda_{22},\\ p_{10}= & \lambda_{6}+\lambda_{7}+\lambda_{9}+\lambda_{10}+\lambda_{13}+\lambda_{14}\\ & +\lambda_{15}+\lambda_{16}+\lambda_{19}+\lambda_{20}+\lambda_{21},\\ p_{11}= & \lambda_{3}+\lambda_{11}+\lambda_{12}+\lambda_{13}+\lambda_{14}+\lambda_{17}\\ & +\lambda_{18}+\lambda_{19}+\lambda_{20}+\lambda_{22}. \end{array}$$

Note that, for all configurations, $p_{a}=\lambda_{1}+\lambda_{2}+\lambda_{3}\leq p_{a^{\prime }}$, implying that, whenever a is true, $a^{\prime }$ must be true, as well.

The Specker bug logic depicted in Figure 4a contains 13 propositions forming seven contexts {a,1,2}, {2,3,4}, {4,5,b}, {b,6,7}, {7,8,9}, {9,10,a} and {3,8,11}, allowing 14 (separating and unital) two-valued states:

(A5) $$\begin{array}{cc} p_{a}= & \lambda_{1}+\lambda_{2}+\lambda_{3},\\ p_{b}= & \lambda_{6}+\lambda_{13}+\lambda_{14},\\ p_{1}= & \lambda_{4}+\lambda_{5}+\lambda_{6}+\lambda_{7}+\lambda_{8}+\lambda_{9},\\ p_{2}= & \lambda_{10}+\lambda_{11}+\lambda_{12}+\lambda_{13}+\lambda_{14},\\ p_{3}= & \lambda_{1}+\lambda_{4}+\lambda_{5}+\lambda_{6},\\ p_{4}= & \lambda_{2}+\lambda_{3}+\lambda_{7}+\lambda_{8}+\lambda_{9},\\ p_{5}= & \lambda_{1}+\lambda_{4}+\lambda_{5}+\lambda_{10}+\lambda_{11}+\lambda_{12},\\ p_{6}= & \lambda_{2}+\lambda_{4}+\lambda_{7}+\lambda_{8}+\lambda_{10}+\lambda_{11},\\ p_{7}= & \lambda_{1}+\lambda_{3}+\lambda_{5}+\lambda_{9}+\lambda_{12},\\ p_{8}= & \lambda_{2}+\lambda_{7}+\lambda_{10}+\lambda_{13},\\ p_{9}= & \lambda_{4}+\lambda_{6}+\lambda_{8}+\lambda_{11}+\lambda_{14},\\ p_{10}= & \lambda_{5}+\lambda_{7}+\lambda_{9}+\lambda_{10}+\lambda_{12}+\lambda_{13},\\ p_{11}= & \lambda_{3}+\lambda_{8}+\lambda_{9}+\lambda_{11}+\lambda_{12}+\lambda_{14}. \end{array}$$

Note that, for all configurations, whenever a is true, **b** is false, and vice versa.

## Appendix E. Truth Assignments, (Quasi)Classical Probabilities on Truth-Implies-Value Indefiniteness Logic in Three Dimensions

Figure 6c depicts 37 propositions {a,b,1,2,3,…,35} in 26 contexts {a,1,2}, {b,2,3}, {4,a,5}, {b,6,7}, ${\left[\left\{7,10,4\right\}\right]}_{\left(a),(c\right)}$, ${\left[\left\{10,12,13\right\}\right]}_{\left(a),(c\right)}$, ${\left[\left\{5,29,23\right\}\right]}_{\left(b),(c\right)}$, ${\left[\left\{13,31,29\right\}\right]}_{\left(b),(c\right)}$, {3,21,23}, {4,28,22}, {22,19,3}, {b,8,9}, {9,11,5}, {28,30,15}, {15,14,11}, {6,33,17}, {17,20,21}, {7,34,27}, {27,26,23}, {22,24,25}, {25,35,9}, {15,17,1}, {13,16,1}, {16,18,19}, {16,32,8} and {25,1,27}, allowing eight (non-separating, non-unital on a, 2, 13, 15, 16, 17, 25, 27) two-valued states whose convex sum yields the following weights:

(A6) $$\begin{array}{cc} p_{a}= & p_{2}=p_{13}=p_{15}=p_{16}=p_{17}=p_{25}=p_{27}=0,\\ p_{b}= & \lambda_{1}+\lambda_{2}+\lambda_{3}+\lambda_{4},\\ p_{1}= & \lambda_{1}+\lambda_{2}+\lambda_{3}+\lambda_{4}+\lambda_{5}+\lambda_{6}+\lambda_{7}+\lambda_{8}=1,\\ p_{3}= & +\lambda_{5}+\lambda_{6}+\lambda_{7}+\lambda_{8},\\ p_{4}= & \lambda_{1}+\lambda_{2}+\lambda_{5}+\lambda_{6},\\ p_{5}= & \lambda_{3}+\lambda_{4}+\lambda_{7}+\lambda_{8},\\ p_{6}= & \lambda_{5}+\lambda_{6}+\lambda_{7},\\ p_{7}= & \lambda_{8},p_{9}=\lambda_{6},p_{22}=\lambda_{4},p_{23}=\lambda_{2},\\ p_{8}= & \lambda_{5}+\lambda_{7}+\lambda_{8},\\ p_{10}= & \lambda_{3}+\lambda_{4}+\lambda_{7},\\ p_{11}= & \lambda_{1}+\lambda_{2}+\lambda_{5},\\ p_{12}= & \lambda_{1}+\lambda_{2}+\lambda_{5}+\lambda_{6}+\lambda_{8},\\ p_{14}= & \lambda_{3}+\lambda_{4}+\lambda_{6}+\lambda_{7}+\lambda_{8},\\ p_{18}= & \lambda_{4}+\lambda_{5}+\lambda_{6}+\lambda_{7}+\lambda_{8},\\ p_{19}= & \lambda_{1}+\lambda_{2}+\lambda_{3},\\ p_{20}= & +\lambda_{2}+\lambda_{5}+\lambda_{6}+\lambda_{7}+\lambda_{8},\\ p_{21}= & \lambda_{1}+\lambda_{3}+\lambda_{4},\\ p_{24}= & \lambda_{1}+\lambda_{2}+\lambda_{3}+\lambda_{5}+\lambda_{6}+\lambda_{7}+\lambda_{8},\\ p_{26}= & \lambda_{1}+\lambda_{3}+\lambda_{4}+\lambda_{5}+\lambda_{6}+\lambda_{7}+\lambda_{8},\\ p_{28}= & \lambda_{3}+\lambda_{7}+\lambda_{8},\\ p_{29}= & \lambda_{1}+\lambda_{5}+\lambda_{6},\\ p_{30}= & \lambda_{1}+\lambda_{2}+\lambda_{4}+\lambda_{5}+\lambda_{6},\\ p_{31}= & \lambda_{2}+\lambda_{3}+\lambda_{4}+\lambda_{7}+\lambda_{8},\\ p_{32}= & \lambda_{1}+\lambda_{2}+\lambda_{3}+\lambda_{4}+\lambda_{6},\\ p_{33}= & \lambda_{1}+\lambda_{2}+\lambda_{3}+\lambda_{4}+\lambda_{8},\\ p_{34}= & \lambda_{1}+\lambda_{2}+\lambda_{3}+\lambda_{4}+\lambda_{5}+\lambda_{6}+\lambda_{7},\\ p_{35}= & \lambda_{1}+\lambda_{2}+\lambda_{3}+\lambda_{4}+\lambda_{5}+\lambda_{7}+\lambda_{8}. \end{array}$$

The logics in Figure 6a,b contain 35 observables in 24 contexts, which are the same as before in Figure 6c, lacking two contexts ${\left[\left\{5,29,23\right\}\right]}_{\left(b),(c\right)}$ and ${\left[\left\{13,31,29\right\}\right]}_{\left(b),(c\right)}$, as well as ${\left[\left\{7,10,4\right\}\right]}_{\left(a),(c\right)}$ and ${\left[\left\{10,12,13\right\}\right]}_{\left(a),(c\right)}$, respectively.

The logic in Figure 6a allows 13 (non-unital on 16) two-valued states whose convex sum yields the following weights:

(A7) $$\begin{array}{cc} p_{a}= & \lambda_{1},\\ p_{b}= & \lambda_{2}+\lambda_{3}+\lambda_{4}+\lambda_{5}+\lambda_{6}+\lambda_{7},\\ p_{16}= & 0,\\ p_{1}= & \lambda_{2}+\lambda_{3}+\lambda_{4}+\lambda_{5}+\lambda_{6}+\lambda_{7}+\lambda_{8}+\lambda_{9}+\lambda_{10}+\lambda_{11},\\ p_{2}= & \lambda_{12}+\lambda_{13},\\ p_{3}= & \lambda_{1}+\lambda_{8}+\lambda_{9}+\lambda_{10}+\lambda_{11},\\ p_{4}= & \lambda_{2}+\lambda_{3}+\lambda_{8}+\lambda_{9}+\lambda_{12},\\ p_{5}= & \lambda_{4}+\lambda_{5}+\lambda_{6}+\lambda_{7}+\lambda_{10}+\lambda_{11}+\lambda_{13},\\ p_{6}= & \lambda_{8}+\lambda_{9}+\lambda_{10}+\lambda_{12},\\ p_{7}= & \lambda_{1}+\lambda_{11}+\lambda_{13},\\ p_{8}= & \lambda_{1}+\lambda_{8}+\lambda_{10}+\lambda_{11}+\lambda_{13},\\ p_{9}= & \lambda_{9}+\lambda_{12},\\ p_{10}= & \lambda_{4}+\lambda_{5}+\lambda_{6}+\lambda_{7}+\lambda_{10},\\ p_{11}= & \lambda_{1}+\lambda_{2}+\lambda_{3}+\lambda_{8},\\ p_{12}= & \lambda_{2}+\lambda_{3}+\lambda_{8}+\lambda_{9}+\lambda_{11},\\ p_{13}= & \lambda_{1}+\lambda_{12}+\lambda_{13},\\ p_{14}= & \lambda_{4}+\lambda_{5}+\lambda_{6}+\lambda_{7}+\lambda_{9}+\lambda_{10}+\lambda_{11}+\lambda_{13},\\ p_{15}= & \lambda_{12},\\ p_{17}= & \lambda_{1}+\lambda_{13},\\ p_{18}= & \lambda_{1}+\lambda_{5}+\lambda_{7}+\lambda_{8}+\lambda_{9}+\lambda_{10}+\lambda_{11},\\ p_{19}= & \lambda_{2}+\lambda_{3}+\lambda_{4}+\lambda_{6}+\lambda_{12}+\lambda_{13},\\ p_{20}= & \lambda_{3}+\lambda_{6}+\lambda_{7}+\lambda_{8}+\lambda_{9}+\lambda_{10}+\lambda_{11},\\ p_{21}= & \lambda_{2}+\lambda_{4}+\lambda_{5}+\lambda_{12},\\ p_{22}= & \lambda_{5}+\lambda_{7},\\ p_{23}= & \lambda_{3}+\lambda_{6}+\lambda_{7}+\lambda_{13},\\ p_{24}= & \lambda_{2}+\lambda_{3}+\lambda_{4}+\lambda_{6}+\lambda_{8}+\lambda_{9}+\lambda_{10}+\lambda_{11}+\lambda_{12},\\ p_{25}= & \lambda_{1}+\lambda_{13},\\ p_{26}= & \lambda_{1}+\lambda_{2}+\lambda_{4}+\lambda_{5}+\lambda_{8}+\lambda_{9}+\lambda_{10}+\lambda_{11},\\ p_{27}= & \lambda_{12},\\ p_{28}= & \lambda_{1}+\lambda_{4}+\lambda_{6}+\lambda_{10}+\lambda_{11}+\lambda_{13},\\ p_{30}= & \lambda_{2}+\lambda_{3}+\lambda_{5}+\lambda_{7}+\lambda_{8}+\lambda_{9},\\ p_{32}= & \lambda_{2}+\lambda_{3}+\lambda_{4}+\lambda_{5}+\lambda_{6}+\lambda_{7}+\lambda_{9}+\lambda_{12},\\ p_{33}= & \lambda_{2}+\lambda_{3}+\lambda_{4}+\lambda_{5}+\lambda_{6}+\lambda_{7}+\lambda_{11},\\ p_{34}= & \lambda_{2}+\lambda_{3}+\lambda_{4}+\lambda_{5}+\lambda_{6}+\lambda_{7}+\lambda_{8}+\lambda_{9}+\lambda_{10},\\ p_{35}= & \lambda_{2}+\lambda_{3}+\lambda_{4}+\lambda_{5}+\lambda_{6}+\lambda_{7}+\lambda_{8}+\lambda_{10}+\lambda_{11}. \end{array}$$

Therefore, whenever a is true, that is, $p_{a}=\lambda_{1}=1$, b has to be false, because $p_{b}=\lambda_{2}+\lambda_{3}+\lambda_{4}+\lambda_{5}+\lambda_{6}+\lambda_{7}=0$.

Conversely, the logic in Figure 6b allows 13 (non-separating on 15/27 and non-unital on 16) two-valued states whose convex sum yields the following weights:

(A8) $$\begin{array}{cc} p_{a}= & \lambda_{1},\\ p_{b}= & \lambda_{1}+\lambda_{2}+\lambda_{3}+\lambda_{4}+\lambda_{5},\\ p_{16}= & 0,\\ p_{1}= & \lambda_{2}+\lambda_{3}+\lambda_{4}+\lambda_{5}+\lambda_{6}+\lambda_{7}+\lambda_{8}+\lambda_{9}+\lambda_{10}+\lambda_{11},\\ p_{2}= & \lambda_{12}+\lambda_{13},\\ p_{3}= & \lambda_{6}+\lambda_{7}+\lambda_{8}+\lambda_{9}+\lambda_{10}+\lambda_{11},\\ p_{4}= & \lambda_{2}+\lambda_{3}+\lambda_{6}+\lambda_{7}+\lambda_{8}+\lambda_{9}+\lambda_{12},\\ p_{5}= & \lambda_{4}+\lambda_{5}+\lambda_{10}+\lambda_{11}+\lambda_{13},\\ p_{6}= & \lambda_{6}+\lambda_{7}+\lambda_{10}+\lambda_{13},\\ p_{7}= & \lambda_{8}+\lambda_{9}+\lambda_{11}+\lambda_{12},\\ p_{8}= & \lambda_{6}+\lambda_{8}+\lambda_{10}+\lambda_{11}+\lambda_{12}+\lambda_{13},\\ p_{9}= & \lambda_{7}+\lambda_{9},\\ p_{11}= & \lambda_{1}+\lambda_{2}+\lambda_{3}+\lambda_{6}+\lambda_{8}+\lambda_{12},\\ p_{13}= & \lambda_{1}+\lambda_{12}+\lambda_{13},\\ p_{14}= & \lambda_{4}+\lambda_{5}+\lambda_{7}+\lambda_{9}+\lambda_{10}+\lambda_{11},\\ p_{15}= & p_{27}=\lambda_{13},\\ p_{17}= & \lambda_{1}+\lambda_{12},\\ p_{18}= & \lambda_{5}+\lambda_{6}+\lambda_{7}+\lambda_{8}+\lambda_{9}+\lambda_{10}+\lambda_{11}+\lambda_{13},\\ p_{19}= & \lambda_{1}+\lambda_{2}+\lambda_{3}+\lambda_{4}+\lambda_{12},\\ p_{20}= & \lambda_{3}+\lambda_{6}+\lambda_{7}+\lambda_{8}+\lambda_{9}+\lambda_{10}+\lambda_{11},\\ p_{21}= & \lambda_{2}+\lambda_{4}+\lambda_{5}+\lambda_{13},\\ p_{22}= & \lambda_{5}+\lambda_{13},\\ p_{23}= & \lambda_{1}+\lambda_{3}+\lambda_{12},\\ p_{24}= & \lambda_{2}+\lambda_{3}+\lambda_{4}+\lambda_{6}+\lambda_{7}+\lambda_{8}+\lambda_{9}+\lambda_{10}+\lambda_{11},\\ p_{25}= & \lambda_{1}+\lambda_{12},\\ p_{26}= & \lambda_{2}+\lambda_{4}+\lambda_{5}+\lambda_{6}+\lambda_{7}+\lambda_{8}+\lambda_{9}+\lambda_{10}+\lambda_{11},\\ p_{28}= & \lambda_{1}+\lambda_{4}+\lambda_{10}+\lambda_{11},\\ p_{29}= & \lambda_{2}+\lambda_{6}+\lambda_{7}+\lambda_{8}+\lambda_{9},\\ p_{30}= & \lambda_{2}+\lambda_{3}+\lambda_{5}+\lambda_{6}+\lambda_{7}+\lambda_{8}+\lambda_{9}+\lambda_{12},\\ p_{31}= & \lambda_{3}+\lambda_{4}+\lambda_{5}+\lambda_{10}+\lambda_{11},\\ p_{32}= & \lambda_{1}+\lambda_{2}+\lambda_{3}+\lambda_{4}+\lambda_{5}+\lambda_{7}+\lambda_{9},\\ p_{33}= & \lambda_{2}+\lambda_{3}+\lambda_{4}+\lambda_{5}+\lambda_{8}+\lambda_{9}+\lambda_{11},\\ p_{34}= & \lambda_{1}+\lambda_{2}+\lambda_{3}+\lambda_{4}+\lambda_{5}+\lambda_{6}+\lambda_{7}+\lambda_{10},\\ p_{35}= & \lambda_{2}+\lambda_{3}+\lambda_{4}+\lambda_{5}+\lambda_{6}+\lambda_{8}+\lambda_{10}+\lambda_{11}+\lambda_{13}. \end{array}$$

Therefore, whenever a is true, that is, $p_{a}=\lambda_{1}=1$, b has to be true, because $p_{b}=\lambda_{1}+\lambda_{2}+\lambda_{3}+\lambda_{4}+\lambda_{5}=\lambda_{1}=1$.

## References

[1] Gleason A.M. (1957). Measures on the closed subspaces of a Hilbert space. J. Math. Mech..

[2] Mermin D.N. (2007). Quantum Computer Science.

[3] Halmos P.R. (1958). Finite-Dimensional Vector Spaces.

[4] Svozil K. (1993). Randomness & Undecidability in Physics.

[5] Dvurečenskij A., Pulmannová S., Svozil K. (1995). Partition Logics, Orthoalgebras and Automata. Helv. Phys. Acta.

[6] Svozil K. (1998). Quantum Logic.

[7] Svozil K. (2005). Logical equivalence between generalized urn models and finite automata. Int. J. Theor. Phys..

[8] Svozil K., Engesser K., Gabbay D.M., Lehmann D. (2009). Contexts in quantum, classical and partition logic. Handbook of Quantum Logic and Quantum Structures.

[9] Svozil K., Burgin M., Calude C.S. (2016). Generalized event structures and probabilities. Information and Complexity.

[10] Svozil K. (2018). Physical [A]Causality. Determinism, Randomness and Uncaused Events.

[11] Chevalier G. (1989). Commutators and decompositions of orthomodular lattices. Order.

[12] Moore E.F., Shannon C.E., McCarthy J. (1956). Gedanken-Experiments on Sequential Machines. Automata Studies.

[13] Schaller M., Svozil K. (1995). Automaton partition logic versus quantum logic. Int. J. Theor. Phys..

[14] Schaller M., Svozil K. (1996). Automaton logic. Int. J. Theor. Phys..

[15] Wright R. (1990). Generalized urn models. Found. Phys..

[16] Svozil K. (2006). Staging quantum cryptography with chocolate balls. Am. J. Phys..

[17] Svozil K. (2014). Non-contextual chocolate ball versus value indefinite quantum cryptography. Theor. Comput. Sci..

[18] Svozil K. (2000). On generalized probabilities: Correlation polytopes for automaton logic and generalized urn models, extensions of quantum mechanics and parameter cheats. arXiv.

[19] Boole G. (1862). On the Theory of Probabilities. Philos. Trans. R. Soc. Lond..

[20] Froissart M. (1981). Constructive generalization of Bell’s inequalities. Il Nuovo Cimento B.

[21] Cirel’son B.S. (1993). Some results and problems on quantum Bell-type inequalities. Hadron. J. Suppl..

[22] Pitowsky I. (1986). The range of quantum probabilities. J. Math. Phys..

[23] Pitowsky I. (1994). George Boole’s ‘Conditions of Possible Experience’ and the Quantum Puzzle. Br. J. Philos. Sci..

[24] Richard J. (1971). Orthomodular lattices admitting no states. J. Comb. Theory Ser. A.

[25] Fukuda K. cdd and cddplus Homepage, cddlib Package cddlib-094h, 2000. http://www.inf.ethz.ch/personal/fukudak/cdd_home/.

[26] Wright R., Marlow A.R. (1978). The state of the pentagon. A nonclassical example. Mathematical Foundations of Quantum Theory.

[27] Kalmbach G. (1983). Orthomodular Lattices (London Mathematical Society Monographs).

[28] Beltrametti E.G., Maçzyński M.J. (1995). On the range of non-classical probability. Rep. Math. Phys..

[29] Klyachko A.A., Can M.A., Binicioğlu S., Shumovsky A.S. (2008). Simple Test for Hidden Variables in Spin-1 Systems. Phys. Rev. Lett..

[30] Bub J., Stairs A. (2009). Contextuality and Nonlocality in ‘No Signaling’ Theories. Found. Phys..

[31] Bub J., Stairs A. (2010). Contextuality in Quantum Mechanics: Testing the Klyachko Inequality. arXiv.

[32] Badzia̧g P., Bengtsson I., Cabello A., Granström H., Larsson J.A. (2011). Pentagrams and Paradoxes. Found. Phys..

[33] Kochen S., Specker E.P. (1965). Logical Structures arising in quantum theory. The Theory of Models, Proceedings of the 1963 International Symposium at Berkeley.

[34] Kochen S., Specker E.P. (1967). The Problem of Hidden Variables in Quantum Mechanics. J. Math. Mech..

[35] Redhead M. (1990). Incompleteness, Nonlocality, and Realism: A Prolegomenon to the Philosophy of Quantum Mechanics.

[36] Pitowsky I. (2003). Betting on the outcomes of measurements: A Bayesian theory of quantum probability. Stud. Hist. Philos. Sci. Part B Stud. Hist. Philos. Mod. Phys..

[37] Pitowsky I., Demopoulos W., Pitowsky I. (2006). Quantum Mechanics as a Theory of Probability. Physical Theory and Its Interpretation.

[38] Belinfante F.J. (1973). A Survey of Hidden-Variables Theories.

[39] Stairs A. (1983). Quantum logic, realism, and value definiteness. Philos. Sci..

[40] (1993). Getting contextual and nonlocal elements-of-reality the easy way. Am. J. Phys..

[41] Pták P., Pulmannová S. (1991). Orthomodular Structures as Quantum Logics. Intrinsic Properties, State Space and Probabilistic Topics.

[42] Navara M., Rogalewicz V. (1991). The pasting constructions for orthomodular posets. Math. Nachr..

[43] Johansen H.B. (1994). Comment on Getting contextual and nonlocal elements-of-reality the easy way. Am. J. Phys..

[44] Vermaas P.E. (1994). Comment on Getting contextual and nonlocal elements-of-reality the easy way. Am. J. Phys..

[45] Cabello A., Portillo J.R., Solís A., Svozil K. (2013). Minimal true-implies-false and true-implies-true sets of propositions in noncontextual hidden variable theories. arXiv.

[46] Svozil K. (2009). Quantum Scholasticism: On Quantum Contexts, Counterfactuals, and the Absurdities of Quantum Omniscience. Inf. Sci..

[47] Cabello A. (1994). A simple proof of the Kochen-Specker theorem. Eur. J. Phys..

[48] Cabello A. (1996). Pruebas Algebraicas de Imposibilidad de Variables Ocultas en Mecánica Cuántica. Ph.D. Thesis.

[49] Tkadlec J. (1998). Greechie diagrams of small quantum logics with small state spaces. Int. J. Theor. Phys..

[50] Svozil K., Tkadlec J. (1996). Greechie diagrams, nonexistence of measures in quantum logics and Kochen–Specker type constructions. J. Math. Phys..

[51] Abbott A.A., Calude C.S., Svozil K. (2015). A variant of the Kochen-Specker theorem localising value indefiniteness. J. Math. Phys..

[52] Pitowsky I. (1982). Substitution and Truth in Quantum Logic. Philos. Sci..

[53] Hardy L. (1992). Quantum mechanics, local realistic theories, and Lorentz-invariant realistic theories. Phys. Rev. Lett..

[54] Hardy L. (1993). Nonlocality for two particles without inequalities for almost all entangled states. Phys. Rev. Lett..

[55] Boschi D., Branca S., De Martini F., Hardy L. (1997). Ladder Proof of Nonlocality without Inequalities: Theoretical and Experimental Results. Phys. Rev. Lett..

[56] Cabello A., García-Alcaine G. (1995). A hidden-variables versus quantum mechanics experiment. J. Phys. A Math. Gen. Phys..

[57] Cabello A., Estebaranz J.M., García-Alcaine G. (1996). Bell-Kochen-Specker theorem: A proof with 18 vectors. Phys. Lett. A.

[58] Cabello A. (1997). No-hidden-variables proof for two spin- particles preselected and postselected in unentangled states. Phys. Rev. A.

[59] Chen J.L., Cabello A., Xu Z.P., Su H.Y., Wu C., Kwek L.C. (2013). Hardy’s paradox for high-dimensional systems. Phys. Rev. A.

[60] Cabello A., Badziag P., Terra Cunha M., Bourennane M. (2013). Simple Hardy-Like Proof of Quantum Contextuality. Phys. Rev. Lett..

[61] Pitowsky I. (1998). Infinite and finite Gleason’s theorems and the logic of indeterminacy. J. Math. Phys..

[62] Hrushovski E., Pitowsky I. (2004). Generalizations of Kochen and Specker’s theorem and the effectiveness of Gleason’s theorem. Stud. Hist. Philos. Sci. Part B Stud. Hist. Philos. Mod. Phys..

[63] Abbott A.A., Calude C.S., Conder J., Svozil K. (2012). Strong Kochen-Specker theorem and incomputability of quantum randomness. Phys. Rev. A.

[64] Abbott A.A., Calude C.S., Svozil K. (2014). Value-indefinite observables are almost everywhere. Phys. Rev. A.

[65] Pitowsky I. (1983). Deterministic model of spin and statistics. Phys. Rev. D.

[66] Meyer D.A. (1999). Finite precision measurement nullifies the Kochen-Specker theorem. Phys. Rev. Lett..

[67] Schrödinger E. (1935). Die gegenwärtige Situation in der Quantenmechanik. Naturwissenschaften.

[68] London F., Bauer E. (1983). The Theory of Observation in Quantum Mechanics. Quantum Theory and Measurement.

[69] Svozil K. (2004). Quantum information via state partitions and the context translation principle. J. Mod. Opt..

[70] Howard D. (2004). Who Invented the “Copenhagen Interpretation”? A Study in Mythology. Philos. Sci..

[71] Bohr N. (1928). The quantum postulate and the recent development of atomistic theory. Nature.

[72] Von Neumann J. (1996). Mathematische Grundlagen der Quantenmechanik.

[73] Zeilinger A. (1999). A Foundational Principle for Quantum Mechanics. Found. Phys..

[74] Myrvold W.C. (2011). Statistical mechanics and thermodynamics: A Maxwellian view. Stud. Hist. Philos. Sci. Part B Stud. Hist. Philos. Mod. Phys..

[75] Specker E. (1960). Die Logik nicht gleichzeitig entscheidbarer Aussagen. Dialectica.

[76] Kochen S., Specker E.P. (1965). The calculus of partial propositional functions. Proceedings of the 1964 International Congress for Logic, Methodology and Philosophy of Science.

[77] Valentini A. (2018). The de Broglie-Bohm Pilot-Wave Theory.

[78] Ferrari G.R.F., Plato (2000). The Republic.

[79] Tkadlec J. (2017). Personal communication.

